# Mule deer spatial association patterns and potential implications for transmission of an epizootic disease

**DOI:** 10.1371/journal.pone.0175385

**Published:** 2017-04-07

**Authors:** María Fernanda Mejía-Salazar, Anne W. Goldizen, Clementine S. Menz, Ross G. Dwyer, Simon P. Blomberg, Cheryl L. Waldner, Catherine I. Cullingham, Trent K. Bollinger

**Affiliations:** 1 Western College of Veterinary Medicine, University of Saskatchewan, Saskatoon, Saskatchewan, Canada; 2 School of Biological Sciences, University of Queensland, Brisbane, Queensland, Australia; 3 Department of Biological Sciences, University of Alberta, Edmonton, Alberta, Canada; 4 Canadian Wildlife Health Cooperative (CWHC), Saskatoon, Saskatchewan, Canada; University of Missouri Columbia, UNITED STATES

## Abstract

Animal social behaviour can have important effects on the long-term dynamics of diseases. In particular, preferential spatial relationships between individuals can lead to differences in the rates of disease spread within a population. We examined the concurrent influence of genetic relatedness, sex, age, home range overlap, time of year, and prion disease status on proximal associations of adult Rocky Mountain mule deer (*Odocoileus hemionus hemionus*) in a chronic wasting disease endemic area. We also quantified the temporal stability of these associations across different sex, age, and disease status classes. We used three years of high frequency telemetry data from 74 individuals to record encounters within 25 m of each other, and to calculate seasonal home range overlap measured by volume of intersection (VI). The strength of pairwise spatial association between adult mule deer was independent of genetic relatedness, age and disease status. Seasonal variation in association strength was not consistent across years, perhaps due to annual changes in weather conditions. The influence of home range overlap on association strength varied seasonally, whereby associations were stronger in pre-rut and fawning than in the rest of the seasons. The sexes of individuals also interacted with both VI and season. At increasing levels of VI, associations were stronger between females than between males and between females and males. The strongest associations in pre-rut were between males, while the strongest in rut were between females and males. The temporal stability of associations was markedly dependant on the sex and the diagnosis of the associating pair. Our findings highlight the importance of considering concurrent effects of biological and environmental factors when seeking to understand the role of social preference in behavioural ecology and disease spread. Applying this knowledge in epidemiological modelling will shed light on the dynamics of disease transmission among mule deer.

## Introduction

The likelihood and duration of associations between individuals are influenced by social structure, inter-group mixing and the size and composition of social groups [1, 2]. As well as influencing the transfer of information throughout a population, the properties of such associations can also affect the rate of spread of infectious diseases [3]. For social species, epidemiological models that assume all hosts have equal probability of association and disease transmission, and that ignore seasonal variation, are no longer considered suitable for the study of complex diseases [4, 5]. To enhance models to guide disease control, studies are needed to quantify the extent to which individuals choose whom to associate with and factors relating to these choices.

Chronic wasting disease (CWD) affects farmed and free-ranging mule deer (*Odocoileus hemionus*) and other cervids in USA and Canada, and most recently free-ranging reindeer (*Rangifer tarandus tarandus*) and moose *(Alces alces*) in Norway. It is a fatal, neurodegenerative, contagious prion disease that is expected to reduce mule deer population sizes [6, 7]. Furthermore, it is proving extremely difficult to eradicate once established within wild populations [8]. The complexity of this disease is due to its transmission through both animal-to-animal contact and through the environment, its lengthy infectious period (>1.5 years), and the persistence of prions in the environment for at least 2.5 years [9, 10]. These factors highlight the need for detailed information on mule deer social behaviour and the dynamics of prions in the environment [5, 11] to parameterise dynamic disease models and inform cervid population management programs. The validity of CWD transmission model outcomes is reliant on accurate parameter estimates that describe deer sociality. While there have been relevant studies done on association patterns among female white-tailed deer (*Odocoileus virginianus*) [4, 12] and their home range establishment [13], it is important to collect data specific to mule deer and to both sexes.

Several factors are known to relate to how individuals socialise. When associations are defined based on two individuals being in the same area, a correlation between home range overlap and spatial association strength is expected. However, associations are not driven solely by home range overlap, but also by complex preferences and avoidances (e.g. [14]). Kin-biased associations in various taxa respond flexibly to changes in ecological context, such as local demography and resource abundance [15]. This is probably why genetic relatedness sometimes correlates with association patterns (e.g. [14, 16]) and sometimes does not (e.g. [17, 18]). Among cervids, red deer (*Cervus elaphus*) preferentially associate with kin [19], while genetic relatedness does not determine social structure of elk (*Cervus canadensis*) [20].

Sex and age of the individuals, as well as time of the year, affect the number, type and duration of relationships [21], (e.g. [22]). Disease can also influence social relationships through strategies that restrict pathogen spread, such as behavioural immunity [23] and sickness behaviour [24]. For example, deer infected with CWD have a reduced likelihood of being found in groups [25], probably as a result of behavioural changes caused by brain injury (e.g. diminished alertness, and ataxia) [26]. The clinical phase lasts from a few weeks to about four months under experimental conditions [27] and from a few months to a year based on our field observations (Mejía-Salazar, unpublished data). Studies on sociality that consider the concurrent effects of home range overlap, kinship, and seasonality, as well as life history characteristics, are therefore necessary to understand the role of preference in social organisation and in the dynamics of disease transmission.

The social life of mule deer is characterized by decisions that change dynamically over time, because ecological context, and group size and composition change on a daily basis [25]. Mule deer have a marked right-skewed distribution of group sizes [25] with obvious seasonality driven by environmental conditions and reproductive behaviour [25, 28–31]. The largest mixed-sex groups are observed in winter, while the smallest are seen during the fawning period [25, 29]. In our study area, open flat habitat is associated with larger groups and a greater frequency of close proximity events (deer within 25 m of each other), while rugged terrain is used by many individuals in small groups [25, 28].

Our first aim was to determine whether a range of factors, including sex, age, CWD status, spatial overlap, genetic relatedness and time of the year, all concurrently influenced the strength of pairwise associations. Our second aim was to test for sex, age and CWD status differences in the temporal stability of spatial associations. To answer these questions, we used spatial and genetic data to investigate patterns of associations among pairs of mule deer in a CWD endemic area. Our findings can serve to clarify aspects of cervid social behaviour that in turn can complement future epidemiological modelling to guide CWD management strategies.

## Methods

### Study population

**Fig 1:**
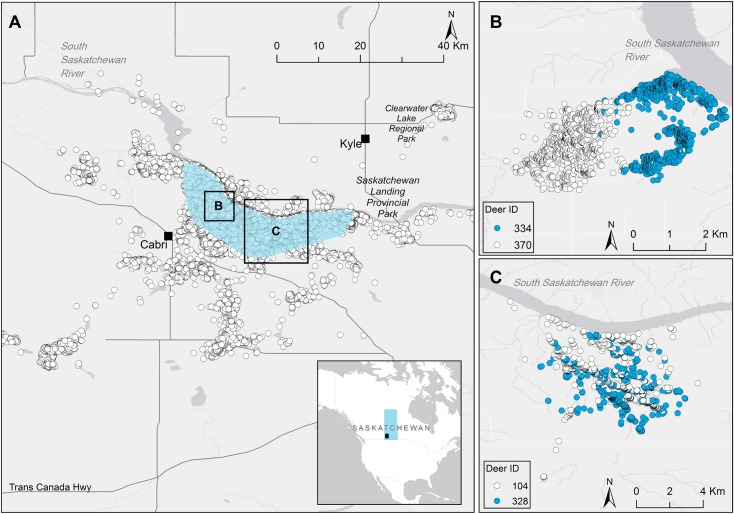
Representation of the study area, and examples of preference and avoidance between individuals. (A) Detail: a map of North America with Saskatchewan in a blue rectangle and the approximate location of the study area in a black rectangle. Map: a close-up of the study area. The vast majority of radio-collared deer moved within a core area (blue polygon) that measured approximately 258 km^2^. Seven individuals relocated to small areas outside the core area for few weeks in a year; the sum of those regions was 44 km^2^. (B) Example of two adult females showing preference for one another during pre-rut in 2010. Even though the volume of intersection (VI) of their home ranges was only 2%, their association index was 1. This means that they were within 25 m of one another at least once a day, every day during that season. (C) Example of two adult males showing avoidance during rut in 2011. This means that despite a VI of 53%, they were not found in close proximity at any time during that season (association index = 0). The maps were created using ArcGIS® software by ESRI [35]; position and arrangement of road networks were obtained from Statistics Canada [36].

The study was conducted between April 2009 and March 2012 in Antelope Creek (50.66°N, 108.27°W), a rural area within the mixed grassland ecoregion in southern Saskatchewan, Canada. The size of the core study area was defined by the movement of radio-collared deer, and was approximately 258 km^2^ (Fig 1A). The north section of the area is limited by the South Saskatchewan River and is characterized by a network of coulees with rugged terrain and natural vegetation. This network is surrounded by predominantly agricultural cropland. The climate is semiarid with long and rigorous winters with mean extreme maximum and minimum temperatures of 35.1°C and -34.2°C, respectively [32]. The population of mule deer in the study area was estimated to range from 322 to 422 mule deer in 2007 and 2009, and was mostly (67%) non-migratory [33], with a mean group size of 3.5 (SD = 3.7, range = 1 to 29) and a typical group size of 7.3 (95% CI = 6.8 to 8.1) [25]. In Antelope Creek, CWD was first recognized in an elk farm in 1998, and then it was detected in wild mule deer in 2000. The prevalence in adult mule deer has substantially increased in Saskatchewan since 2004 [34].

### Data collection

Our animal handling protocol adhered to the Canadian Council on Animal Care guidelines for humane animal use and was approved by the University of Saskatchewan’s Animal Research Ethics Board (Permit number 20050135). Permits to conduct research within private land of the study area were obtained verbally from land owners. A permit to conduct research within the Cabri Regional Park (50.66824°N -108.26791°W) was obtained from The Saskatchewan Regional Parks Association.

We captured mule deer in February or March of 2009, 2010 and 2011 using a helicopter and net-gun [37], or less frequently using Clover traps [38]. Upon capture, deer were chemically immobilized as described by Silbernagel et al. [28]. We collected a 5 mm ear biopsy for genetic analysis. We aged deer based on tooth wear and replacement [39]. Deer are usually classified as adults at 24 months of age [40]; however, we classified deer as adults from 21 months old, as that was their age when we did our annual captures. Immunohistochemical (IHC) staining on palatine tonsil and sometimes rectal biopsies obtained during capture were used to classify the CWD status of live individuals in one of three categories: negative (no immunolabeling in at least 5 lymphoid follicles), positive (immunolabeling in any number of lymphoid follicles), or inconclusive (fewer than 5 lymphoid follicles in the sample) [41, 42]. A minimum of 5 lymphoid follicles in the sample were required to ensure >95% probability of an accurate test [43]. By using this criterion, we reduced the chance of misclassifying a deer as negative due to repeated sampling or increasing age [43, 44]. When the diagnosis was inconclusive, re-cuts of the tonsil and in some cases of rectal biopsies were tested until a final diagnosis was achieved. In spite of retesting, 5 individuals were diagnosed as inconclusive for one year, but were negative when resampled at a later date. These deer were considered negative for all the years previous to the CWD negative result. For dead animals, IHC was performed on portions of obex, tonsil and/or retropharyngeal lymph node. Known positive deer were not removed from the population because data obtained from long-term intensive monitoring of both infected and healthy individuals was considered to be more valuable for epidemiological purposes than removing a few infected individuals from this population. Disease and population control programs in the study area, based on hunting, were not interrupted during the duration of this project and some collared deer were shot by hunters.

Adults were fitted with global positioning system (GPS) radio-collars (Lotek Wireless, Ontario, Canada) that were programmed to record position at predetermined 2 h intervals throughout the day (all at the same times). We released the deer close to their original capture location. Each year, CWD negative deer were re-captured, re-tested, and fitted with a new collar. Deer that tested positive were not re-captured. Data from defective collars were not included in the analyses (Table 1).

**Table 1:** Number of adult (≥ 21 months old) mule deer fitted with GPS collars, by sex and CWD diagnosis. Of 96 unique individuals (some deer were studied in more than one year), data from 74 were suitable for analyses of association strength, given available data on their genetics and locations.

	2009	2010	2011
Positive females	5	4	14
Negative females	21	14	12
Positive males	4	3	14
Negative males	12	4	20
Total	42	25	60

### Data analysis

#### Defining study periods

A study year was defined by the capture period, and ran from 1 April of one year to 31 March of the next year. For this study, we included 3 years of data from 1 April 2009 to 31 March 2012, with each year divided into 5 seasons (Table 2) [25, 28, 32, 45].

**Table 2:** Seasons as defined according to mule deer biology and weather.

Season name (abbreviation)	Start and end dates	Description
Late gestation (LG)	1 Apr-15 May	Males separate from females and fawns, and form bachelor groups (the largest in the year). The frequency of cold temperatures (0 to 10°C) starts to decrease.
Fawning (F)	16 May-31 Jul	Smallest groups in the year. Fawns from the previous year start separating from their mother’s group. Females give birth in synchrony, isolating themselves to give birth. Ambient warm temperatures (24 to 29°C) reach their highest point of the year.
Pre-rut (PR)	1 Aug-31 Oct	Females without fawns are in small groups. Mothers with fawns start to allow other sex and age classes to join them. More frequent male-male interactions in preparation for rut. Warm ambient temperatures start to decrease.
Rut (R)	1 Nov-15 Dec	Oestrus begins. Males become more active to gain breeding access. Freezing ambient temperatures (-10 to 0°C) are the most common.
Early gestation (EG)	16 Dec-31 Mar	The largest mixed-sex herds of the year are formed. Largest female groups of the year. Snow fall peaks and the ambient temperatures are the lowest of the year (below -10°C).

#### Age, sex and disease status

We classified adult mule deer based on age (young adult if 1.8 to 3 years old, or old adult if >3 years old), sex (female or male), and CWD diagnosis (negative, positive, or sick). Deer were assumed to be positive for CWD from the first day of the season in which the sample was taken. For example, if a sample taken on 20 Feb was positive, that deer was considered positive since 16 Dec. All deer were directly observed at least once a month (positive deer at least twice a month) and were considered sick from the moment they showed clear clinical signs of CWD, which included some or all of the following: drooping ears and head, laterally wide feet stance, hocks touching, protruding ribs and ischial tuberosities, reduced alertness, and difficulty in following a group or standing or eating [46]. Deer were considered negative until the season in which they tested positive.

#### Genetic relatedness

Genomic DNA was extracted from ear biopsies of most captured individuals. We genotyped each sample at 16 microsatellite loci following Cullingham et al. [47]. Samples with ≥ 3 missing loci were discarded. Pairwise relatedness measures were estimated for 211 mule deer in the study area, including deer from other research projects, in SPAGeDi version 1.4 [48] using the estimator of Queller & Goodnight [49]. This genetic relatedness coefficient (range from -1 to 1) is an unbiased estimate of relatedness based on the population’s allele frequencies. A positive value indicates that a pair is more related, and a negative value indicates that a pair is less related, than average for the sampled population [49].

#### Analysis of associations

Analysis of pairwise associations was based on radio-telemetry data from adult mule deer with GPS collars (96 different deer in the 3 years) (Table 1). Two individuals were considered associated if they were simultaneously located within 25 m from each other. We chose this threshold to account for collar error, which was 10.3 m on average (n = 16, range = 5.0 to 19.6 m) [28]. To obtain a list of all associated dyads per GPS-fix (i.e. every 2 hours) per season, we used the adehabitatHR package [50, 51] for R. Associations detected within the first 2 weeks after capture were discarded to exclude data that might be affected by behavioural changes related to capture. For the final dataset, deer were considered to be associated on a particular day if they were associated during at least one of the 2-hourly fixes. We calculated the strengths of dyadic associations among all pairs for each season using data files in linear mode and sampling periods of 1 day in SOCPROG 2.6 [52, 53]. As new deer would enter the sample with each collar deployment, while others left due to death or collar failure, we used the social affinity index as the association index because this measure helps to control for such demographic changes (pg. 98 in [21]) [54]. We calculated social affinity indices with this formula: *x*/Min{(*x* + *y*AB + *y*A),(*x* + *y*AB + *y*B)}, where *x* is the number of sampling days that A and B were observed together; Min stands for minimum and indicates that *x* will be divided by whichever of the 2 terms separated by the comma is smaller; *y*A is the number of sampling days that A was observed without B, *y*B is the number of sampling days that B was observed without A, and *y*AB is the number of sampling days in which A and B were both observed, but not together (pg. 98 in [21]), [54]. The index ranges from 0 (deer never detected together within the season) to 1 (deer detected together every day of the season).

#### Calculation of volume of intersection (VI)

We estimated home range size for each individual for each season using the Brownian bridge kernel method [55, 56] in adehabitatHR package [50, 57] for R. This method estimates the utilization distribution of an animal when locations are autocorrelated, which was the case for our data collected every 2 hours. It considers both the positions of the fixes, the path travelled by the animal, and the time dependence between successive fixes [55, 56]. For the final calculations, we excluded fixes within the first 2 weeks after capture, and sequential fixes indicating a movement velocity greater than 2 km/h (an unlikely rate of movement for this species [33, 58]). We used an approach defined by Horne et al. [56] to find the maximum likelihood estimation of the parameter sig1 (a parameter to compute the variance of the position) for every deer and every season. We used a sig2 (location error) of 10 m based on collar accuracy [28], and a grid resolution of 200 m. The areas of home range overlap between deer pairs per season were calculated using adehabitatHR package [57] for R following the volume of intersection (VI) method [59]. This provides a single measure of the VI between the Brownian bridge kernel home ranges of two individuals, per season. The VI ranges from 0 (when two home ranges have no overlap) to 1 (when two individuals have identical utilization distributions). As this method overcomes assumptions about random space use within a home range [59], it produces more biologically meaningful results than simpler measures of areas of overlap.

#### Aim 1: Factors relating to association strength

To investigate the effects of multiple factors on association strength, we built a dataset of all seasonal pairwise associations, excluding records in which both the association index and VI were 0, and records with VI < 0.01 (10 of those 1272 records had association indices of 0.02 to 0.2; the rest had values of 0). We did this to exclude cases in which deer did not have the opportunity to associate. Using the dataset in S1 Appendix, worksheet association_strength_data, we built a set of 15 *a priori* linear mixed effects models using our knowledge of mule deer biology and behaviour (Table 3). The response variable was social affinity index, which was arcsined square-root transformed (asin (sqrt(index)). All inferences were made on the transformed data. The predictor variables included different combinations of season, year, sex (sexes of the pair; e.g. FF for a pair of females), age (ages of the pair; e.g. YY for a pair of young adults), diagnosis (CWD diagnoses of the pair; e.g. SP for a pair of a sick and a positive deer), genetic relatedness, VI, and biologically meaningful 2-way interaction effects. We assigned each dyad a unique code, and treated dyad as a random effect. We used lme4 [60] and MuMIn [61] packages for R [62] to fit these models. Estimates of the relative importance of each predictor variable were calculated by summing the Akaike weights across all models in the set in which the variable occurred (pg. 167 in [63]). We selected the best model based on the Akaike weights (sum is just ≥0.95), and the delta Δ_*p*_ (Δ_*p*_ is ≤ 5; Δ_*p*_ is the difference between AICc from the best model and the next model) (pg. 168 in [63]). We obtained least squares means (LSmeans) to evaluate statistical differences (*P* < 0.05) using Bonferroni correction for multiple comparisons [64]. We report results on back-transformed association indices as predicted by the best model.

**Table 3:** Summary of 15 *a priori* models of mule deer association indices in Antelope Creek, Saskatchewan, Canada.

	Model ID														
Predictor variable	1	2	3	4	5	6	7	8	9	10	11	12	13	14	15
Season	X	X	X	X	X	X	X	X	X	X	X	X	X	X	X
Year	X	X	X	X	X	X	X	X	X	X	X	X	X	X	X
Sex	X	X	X	X			X	X		X	X	X		X	X
Diagnosis	X		X		X			X		X	X		X		
VI	X		X			X	X	X	X		X	X	X	X	X
Age	X			X	X	X					X	X	X		
Relatedness	X						X	X		X					
Season*year	X	X	X	X	X	X	X	X	X	X	X	X	X	X	X
Season*diagnosis	X		X		X			X		X	X				
Sex*relatedness	X						X	X		X					
Season*VI	X		X			X	X	X	X		X	X	X	X	X
Diagnosis*sex	X		X					X		X	X				
VI*diagnosis	X		X					X			X				
Sex*age												X			
Sex*season												X		X	X
Sex*VI															X

Predictor variables considered in each model are indicated with an X.

Terms with an * are 2-way interactions. VI = volume of intersection.

#### Aim 2: Temporal patterns of associations

To test for sex, age and CWD status differences in temporal stability of associations among adult mule deer, we analysed lagged association rates (LARs) in SOCPROG 2.6. LARs are estimates of the probability of association *t* time units after a previous association, averaged over all associations [21, 65]. We only included individuals for which we had continuous GPS-telemetry data from April 1^**st**^ 2011 to March 31^**st**^ 2012 (i.e. study year 2011). Of the 44 mule deer included in the analyses, there were 21 females and 23 males, 16 young adults and 28 old adults, and 12 CWD-negatives and 32 CWD-positives. CWD-positives were those that started the year with a positive diagnosis (n = 17) plus those that became positive during the year (n = 15). For this analysis, we did not classify deer as sick, as none showed clinical signs for the whole year. We investigated the between- and within-class lagged association rates (pg. 89 in [21]), for these class combinations: female-female (FF, n = 50), male-male (MM, n = 122) and female-male (FM, n = 172) pairs; old-old (OO, n = 124), young-young (YY, n = 45) and old-young (OY, n = 175) pairs; and positive-positive (PP, n = 190), negative-negative (NN, n = 20) and positive-negative (PN, n = 134) pairs. LAR_2011_data and LAR_attributes (S1 Appendix) were used to carry out the analyses between classes in SOCPROG 2.6. We set the sampling period as “date” (i.e. 1 day), defined associations as “grouped in sampling period”, and entered the class variables (e.g. LARs from females to males). Then, a set of 7 mathematical models was fitted simultaneously to the observed LARs (Table C in S2 Appendix). These models were of the exponential family and were composed of all, one, or any meaningful combination of three components: rapid disassociations (associations lasting 1 day at most), casual acquaintances (associations that decay over time; their rate of decay is given by a1 and the average duration is approximated from the exponent of the exponential function, e.g. 1/a1, in days), and constant companionships (associations that do not decay or increase over time; their duration is interpreted within the context of the study period, in this case 1 year) [65]. For each of the class pairs, the best fitting and most parsimonious model was indicated by the smallest quasi-Akaike information criterion (QAIC). If the difference between the QAIC of any other model and the best model, ΔQAIC, went from 0 to 2, then there was substantial support for that other model [66]. The estimates of precision for the association rates and their durations were calculated with a Jackknife procedure over 3-day periods, and in some cases (MM, YY and PP) over 30, 45 and 10-day periods, respectively, to obtain better estimates [53]. LARs were compared to a null association rate (NAR), the expected LAR if animals had associated randomly [66].

## Results

### Aim 1: Factors affecting association strength

Between 2009 and 2011, 96 adult mule deer were fitted with GPS collars collecting data every 2 h, 24 h a day. Of these, data from 74 deer were suitable for analyses, given available paired data on both their genetics and locations. Association indices among these 74 deer were on average 0.12 (SE = 0.004, range 0 to 1). Most (58.7%) pairs’ association indices were 0, 23.9% were 0.01 to 0.25, 15.8% were 0.26 to 0.99, and 1.6% were 1. Genetic relatedness was on average -0.003 (SE = 0.005, SD = 0.15, range -0.4 to 0.6). VI of the population was on average 0.17 (SE = 0.003, range 0.01 to 0.95). Preferences for others (i.e. association indices of 1) occurred with a VI as low as 0.02 (Fig 1B), and avoidances (i.e. association indices of 0) occurred when pairs had a VI as high as 0.53 (Fig 1C).

After fitting the *a priori* models predicting strength among adult mule deer, predictor variables were ranked based on their importance (Table 4), and the models were ranked by AICc (Table 5). Based on the Akaike weights and the delta **Δ**_*p*_, model 15 was clearly superior to the rest (delta = 0, weight = 0.9999) (Table 5). This model included the following statistical significant (all *P* < 0.0001) fixed effects: season, year, VI, sex, and four interaction terms–season*year, season*sex, season*VI and sex*VI (S3 Appendix). The age, CWD status, and genetic relatedness of the pair were not significant (*P* > 0.05) predictors of association strength and their relative importances were very small (Table 4).

**Table 4:** Predictor weights used to assess the relative importance of an individual covariate within a model.

Predictor variable	Predictor weight ^a^	Number of models ^b^
Season	1	15
Year	1	15
Season*year	1	15
VI	1	11
Sex	1	11
Season*VI	1	11
Season*sex	1	3
Sex*VI	1	1
Age	< 0.01	7
Age*sex	< 0.01	1
Diagnosis	< 0.01	7
Diagnosis*season	< 0.01	6
Diagnosis*sex	< 0.01	5
Relatedness	< 0.01	4
Relatedness*sex	< 0.01	4
Diagnosis*VI	< 0.01	4

a = predictor weights calculated by summing the Akaike weights for all models in the a priori set in which the variable occurred. The larger the predictor weight, the greater the importance of that predictor.

b = number of models in which the variable occurred.

* = denote 2-way interaction terms.

**Table 5:** Model selection results for strength of spatial association mixed-effects model analyses. Models are ranked according to Akaike information criterion (AICc) and presented along with the delta (Δ_*p*_, the change in AICc relative to the best model), and Akaike weights. Model 15 had a 99.99% chance of being the best model.

Model ID	DF	logLik	AICc	Delta	Akaike weight
15	34	434.2	-799.7	0.0	0.9999
14	32	423.0	-781.3	18.4	1.0 x 10^−4^
12	38	427.7	-778.3	21.4	2.3 x 10^−5^
13	29	390.1	-721.6	78.0	1.1 x 10^−17^
6	24	382.8	-717.2	82.4	1.3 x 10^−18^
9	22	380.4	-716.5	83.2	8.8 x 10^−19^
7	27	381.7	-708.9	90.7	2.0 x 10^−20^
11	66	420.5	-706.1	93.6	4.7 x 10^−21^
3	64	418.4	-706.1	93.6	4.7 x 10^−21^
8	67	418.8	-700.5	99.1	3.0 x 10^−22^
1	69	420.7	-700.2	99.4	2.6 x 10^−22^
2	19	-523.0	1084.2	1883.9	0
4	21	-522.1	1086.6	1886.2	0
10	57	-502.3	1120.9	1920.6	0
5	44	-526.8	1143.0	1942.7	0

Here, we report multiple comparisons of LSmeans using the Bonferroni correction [64]; the association indices as predicted by the best model are back-transformed. See Tables H, I and J in S4 Appendix for predicted values with 95% CI, and *P*-values, test statistics and degrees of freedom of multiple comparisons.

**Fig 2:**
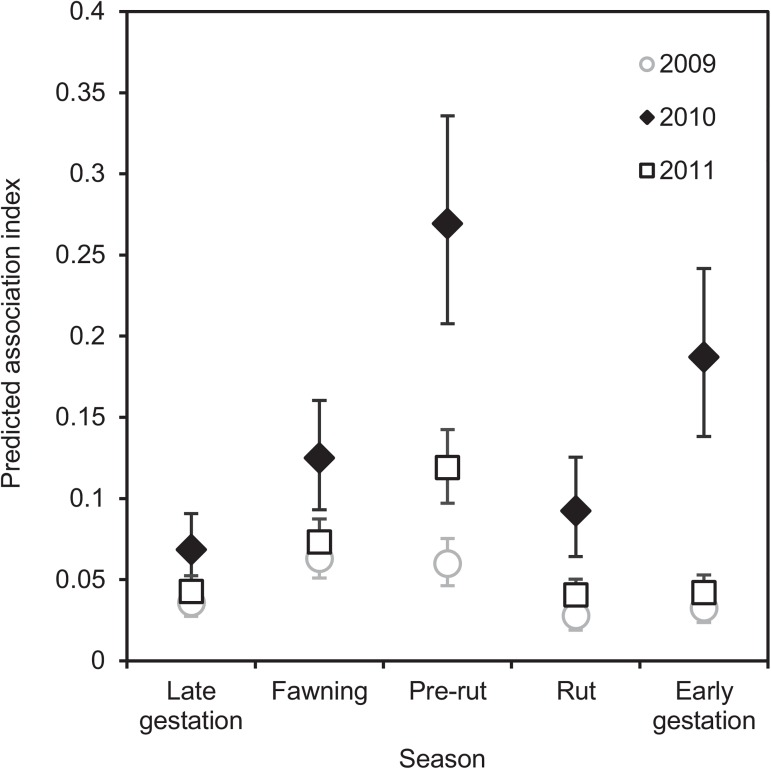
Seasonal predicted pairwise spatial association indices by year among adult mule deer. Error bars are 95% CI. For observed values, see S1 Fig; for values used to generate this graph, see Table H in S4 Appendix.

The interaction between season and year had a significant effect on association strength (*P* < 0.0001) (Fig 2; Table I in S4 Appendix). Association indices between adult mule deer during the pre-rut were significantly different from one year to another (all *P* < 0.0001). Associations were stronger in 2010 than in 2009 in every season (all *P* < 0.02), and were also stronger in 2010 than in 2011 from fawning to early gestation (all *P* < 0.045). During 2009, association indices were significantly stronger during fawning (all *P* < 0.01) and pre-rut (all *P* < 0.045) than in the other three seasons. In 2010, associations were stronger in pre-rut than in any other season (all *P* < 0.0001) except for early gestation (*P* = 0.69), weaker in late gestation than in fawning (*P* = 0.045), and stronger in early gestation than in late gestation (*P* < 0.0001) and rut (*P* = 0.02). In 2011 associations were strongest in pre-rut (all *P* < 0.01), and stronger in fawning than in late gestation (*P* = 0.002), rut (*P* < 0.0001) or early gestation (*P* = 0.002).

**Fig 3:**
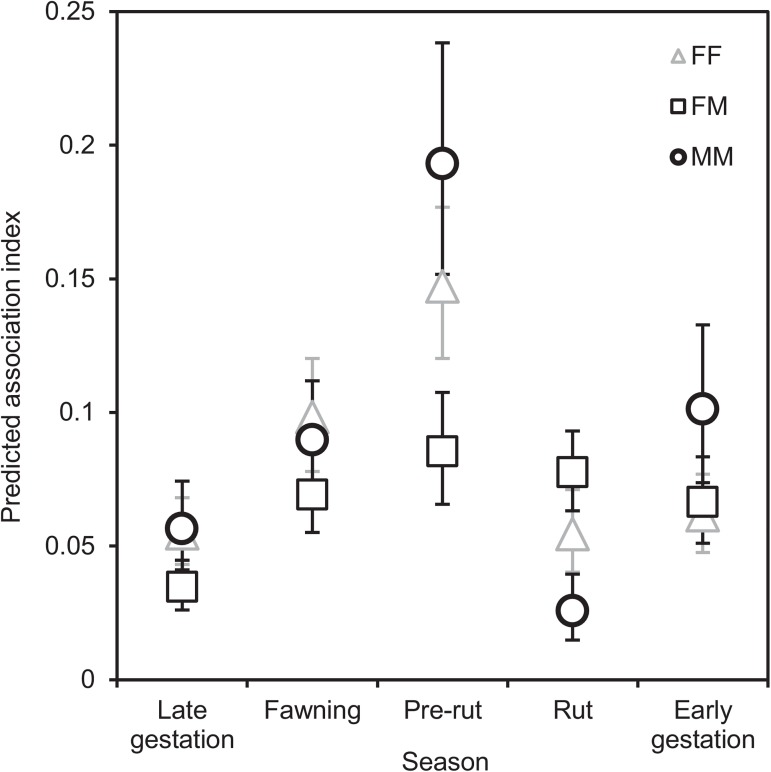
Seasonal predicted pairwise spatial association indices by sex class among adult mule deer. FF are pairs of females, MM are pairs of males, and FM are female-male pairs. Error bars are 95% CI. See S2 Fig for observed values; for values used to generate this graph, see Table H in S4 Appendix.

The interaction between season and sex was also a significant predictor of association strength (*P* < 0.0001) (Fig 3; Table J in S4 Appendix). Pre-rut and rut were the only seasons in which association strength differed significantly among sex classes. In pre-rut, different-sex associations were weaker than same-sex associations (all *P* < 0.002), while during the rut MM associations were significantly (all *P* < 0.045) weaker than FF and FM associations. For each pair class, the strength of the association also varied across seasons: MM associations were weakest in rut (all *P* < 0.03) and strongest in pre-rut (all *P* < 0.01), FF associations were strongest in pre-rut (all *P* < 0.045), and FM associations were weakest in late gestation (all *P* < 0.01).

**Fig 4:**
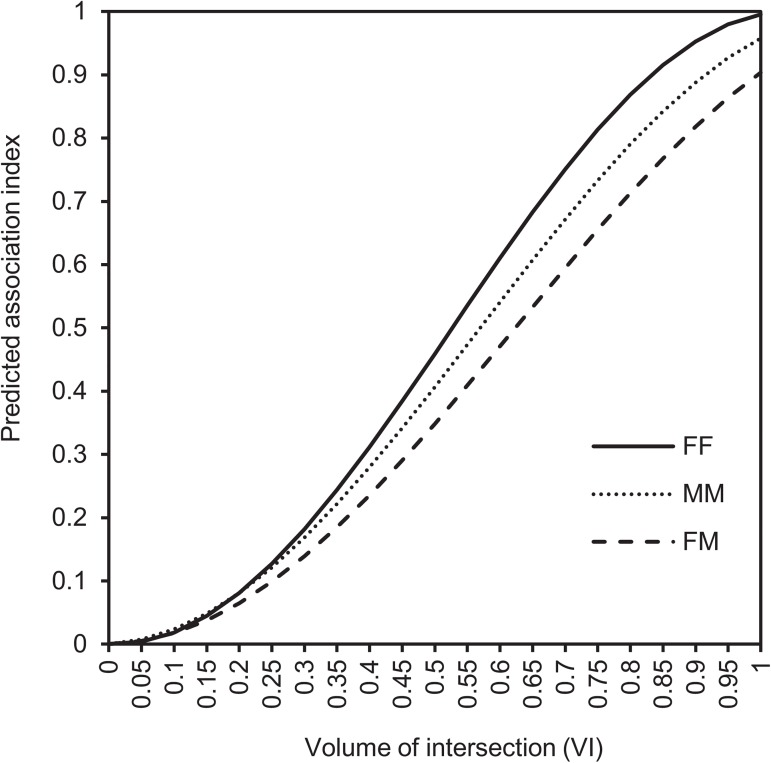
Predicted pairwise spatial association indices by sex class at different levels of volume of intersection (VI) among adult mule deer. FF are pairs of females, MM are pairs of males, and FM are female-male pairs. No confidence intervals (CI) are shown to facilitate graph readability. For CI and observed values, see S3 Fig.

**Fig 5:**
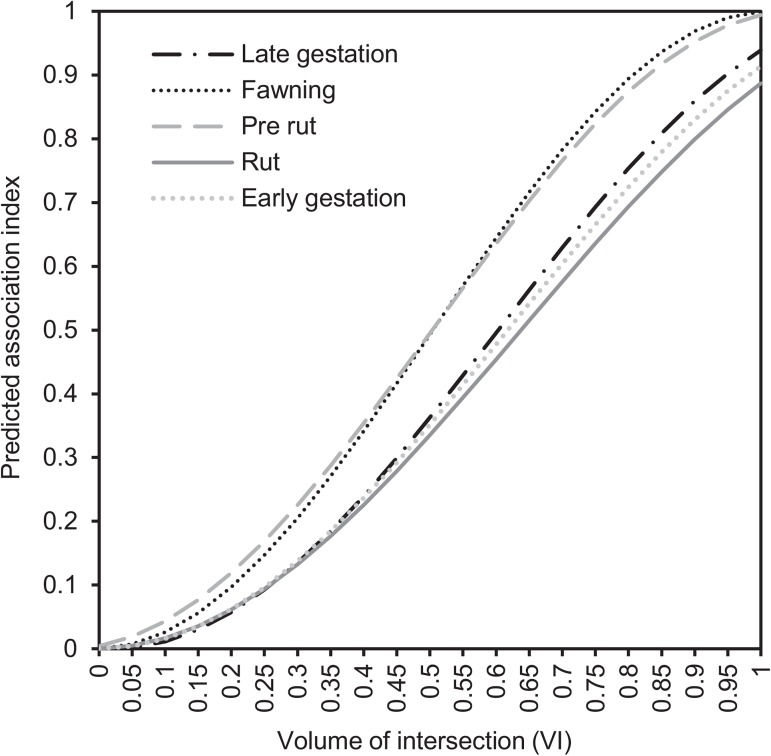
Seasonal predicted pairwise spatial association indices at different levels of volume of intersection (VI) among adult mule deer. No confidence intervals (CI) are shown to facilitate graph readability. For CI and observed values, see S4 Fig.

Sex classes and VI also interacted significantly in their effects on association strength (*P* < 0.0001) (Fig 4;), as did season and VI (*P* < 0.0001) (Fig 5). For all sexes (Fig 4) and all seasons (Fig 5), an increase in VI resulted in an increase in association strength. Notably, at increasing levels of VI, associations were stronger for FF pairs than for MM and FM pairs (Fig 4), and in pre-rut and fawning than in the rest of the seasons (Fig 5).

### Aim 2: Temporal patterns of associations

**Fig 6:**
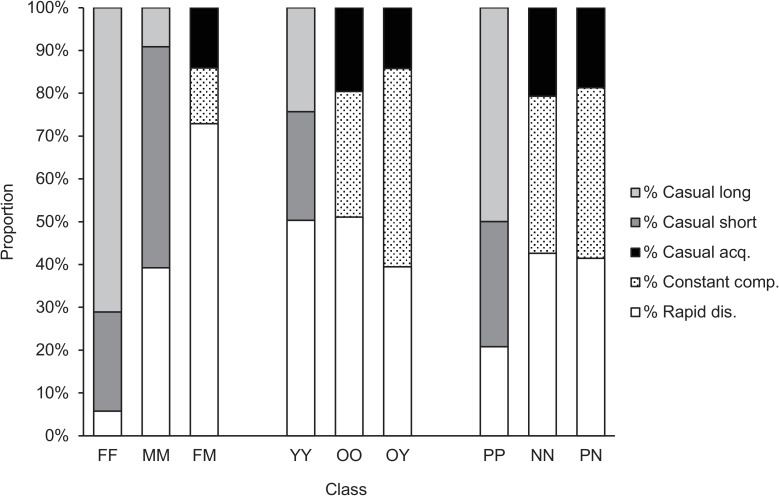
Proportions of components in lagged association rate models among 44 adult mule deer in Saskatchewan, Canada. FF = female-female pairs, MM = male-male pairs, FM = female-male pairs; YY = young-young pairs, OO = old-old pairs, OY = old-young pairs; PP = positive-positive pairs, NN = negative-negative pairs, and PN = positive-negative pairs. Rapid disassociations were associations that lasted the sampling period (i.e. 1 day) at most. In constant companionships, the probability of re-association did not decay or increase over time within the context of the study period (i.e. 1 year). In casual acquaintances, the probability of re-association decayed over time, and their rate of decay was approximated from a1. In some cases, LARs of casual acquaintances decreased over 2 different time scales, one lasting longer (casual long) than the other (casual short). For formulae, and results on durations and SE, see S2 Appendix.

The class LARs among 44 adult mule deer in 2011 were best described by either one of two models: (A) a model containing rapid disassociations, constant companionships and casual acquaintances, or (B) a model containing rapid disassociations and two levels of casual acquaintances, one lasting longer than the other (Fig 6; Table C in S2 Appendix). The LARs, which decreased over time, always remained above the null association rate across all sex, age and CWD status classes (Figures A, B, C, D, E, F, G, H and I in S2 Appendix). Analyses between MM and YY associations produced cyclic-like patterns in the LARs (Figures G and H in S2 Appendix), and also values of SE and duration ranges that were implausible (e.g. 96 days on average ranging from 1 to 1) (Table G in S2 Appendix) despite several tests with different Jackknife levels.

With respect to sex differences in the temporal stability of class associations, the great majority (94.3%) of associations between females disassociated over two different time scales (i.e. two levels of casual acquaintances), with most (71.1%) disassociating after a longer period of association (about 980 days) and 23.2% after 1 to 3 days. Only 5.7% of FF associations lasted no more than 1 day (i.e. rapid disassociations) (Fig 6; Table G in S2 Appendix). In contrast, most (72.9%) of the FM associations lasted no more than 1 day, while the rest either decreased over time (i.e. casual acquaintances) (14.1%), lasting about 17 days, or were stable over the year (i.e. constant companionships) (13%) (Fig 6; Table E in S2 Appendix).

With respect to age differences in the temporal stability of class associations, many (51.1%) associations between older deer (OO) lasted no more than 1 day, while the rest were either stable over the year (29.4%), or decreased over time (19.6%), lasting about 39 days before disassociation occurred (Fig 6; Table E in S2 Appendix). Older and younger deer mainly (46.3%) associated at a constant rate over the year, and less commonly (14.2%) associated as casual acquaintances that were together for about 47 days before disassociating (Fig 6; Table E in S2 Appendix).

Temporal stability also differed with CWD status. Pairs of CWD-negative deer (NN), and of CWD-positive and negative deer (PN), associated similarly (Fig 6). Both cases had similar proportions of their elements and were better described by the model including constant companionships (model A) (Table E in S2 Appendix). In contrast, associations between positive deer (PP) were better described by the model without constant companionships (model B) (Table G in S2 Appendix). Moreover, 79.2% of PP pairs disassociated at two different time scales: 29.3% after about 2 days of association, and 49.9% after about 3 years (Fig 6; Table G in S2 Appendix).

## Discussion

Social behaviours that influence contact rates and the sharing of space in animal species are potentially important factors in information and disease spread within populations [1, 67]. For example, data on association patterns have proven useful in understanding the ecology of diseases that can be transmitted through both direct and environmental contacts, such as tuberculosis in wild animals [3, 68, 69], and CWD in female white-tailed deer [4]. We found that pairwise spatial association patterns of adult mule deer were independent of genetic relatedness, age and CWD status, but seasonal association strength varied with year, sex and home range overlap. We also found important sex and CWD status differences in the temporal stability of spatial associations. By identifying the factors related to individuals’ choices of association partners, we provide empirical data to increase understanding of the possible role of social behaviour in the long-term dynamics of disease transmission among mule deer.

In this study, the strength of associations among mule deer varied among years, with stronger associations in 2010, and a marked peak during the winter (early gestation) of that year. The 2010 pattern may be linked to weather, as the frequency of very cold days (-34 to -10°C) was greater and the mean temperature colder in the winter of 2010 than in 2009 or 2011 (-11 vs -8 and -5°C, respectively) [70]. There was also almost twice as much snow on the ground on a daily basis during rut and early gestation in 2010 than in 2009 (means = 16 vs 9 cm) [70]. In severe winters with decreased temperatures and increased snow depth, escape from predators is hindered [71], forage availability declines and the energetic costs of foraging increase [72]. This forces deer onto the southerly aspects of hills where solar radiation reduces snow cover, resulting in larger winter aggregations.

We also observed seasonal patterns that varied in relation to sex of the associating pair. During pre-rut, associations between males were the strongest and different-sex associations the weakest. Later, in rut, male-male association strength markedly decreased and became weaker than female-female and female-male associations. These are distinctive patterns that are likely driven by mule deer courting and mating behaviour [31]. Prior to females entering oestrus, males establish their dominance using threats and intimidation displays that occur in very close proximity, when contenders circle each other, snort and lick their noses [73], sometimes followed by sparring matches [74]. These behaviours result in more male-male proximity events, and consequently in a peak in male-male spatial association indices in pre-rut. Then, in rut, the frequency and variety of male vocalizations related to courting increase [75], probably to alert other males from a distance and discourage close-contact confrontations [31]. Moreover, male mule deer wander more widely throughout their home ranges during rut to closely follow females to test if they are in oestrus, moving from one female group to the next [76]. Once in oestrus, females allow males to lick their genitals and copulate [76]. These behaviours increase spatial associations between adult females and males, and decrease those between males. In terms of disease, the risk of direct animal to animal transmission between adult males is likely increased in pre-rut, whereas that between adult males and females is probably increased during rut. However, these suggestions require further research, as sharing space does not necessarily translate into a greater frequency of physical contacts among deer (e.g. [77, 78]), and increased spatial association may more accurately translate into increased risk of transmission through sharing contaminated environments.

Genetic relatedness was not an important predictor of spatial associations among adult mule deer in our study area, suggesting that at a very short distance (within 25 m), there is no genetic structuring among these adult mule deer. Similarly, the frequency and duration of proximity instances (within 1.4 m) were not related to genetic relatedness in elk [20]. At a larger spatial scale (km), a study of mule deer [79] also found low levels of genetic structure and limited genetic isolation. However, there were very few highly related adult individuals in our data set (6/982 pairs r > 0.4), therefore, we are unable to determine whether there would be an increased number of associations among close relatives. In contrast, spatially proximate individuals were more genetically related in studies of mule deer [47], white-tailed deer [80–83], Sitka black-tailed deer (*O*. *hemionus sitkensis*) [84], and non-*Odocoileus* cervid species [85–87]. However, the strength of the correlation between kinship and spatial separation or home range overlap varies depending on the set of deer considered in the analysis, and the scale used for measuring spatial distance [88]. It is not surprising that studies that limit analyses to pairs of deer in close proximity (e.g. captured within 1.5 km from each other, with known home range overlap), and that exclude dispersing males and include individuals with high genetic relatedness (e.g. does and fawns), often show a strong association between genetic and spatial distances (e.g. [82]). Although the spatial genetic structure of mule deer is driven by female philopatry and dispersal of males at large spatial scales [47], our data suggest that even at small scales, adults tend to mix freely, suggesting that disease in adults would spread beyond family groups as it should be transmitted similarly among related and unrelated individuals [20, 88].

The greater the VI between pairs of mule deer, the stronger the association they exhibited, irrespective of season and sex class. These findings were expected given that two individuals must be in the same area in order to associate. However, we found cases of apparent preferences (very large association index despite very small VI) and avoidances (very small association index despite large VI), suggesting that mule deer do not associate at random, and that their spatial associations are only partially explained by the extent of their home range overlap, as previously reported in species with fluid fission-fusion dynamics such as eastern grey kangaroos (*Macropus giganteus*) [89], giraffes (*Giraffa camelopardalis*) [14] and eastern water dragons (*Intellagama lesueurii*) [90]. In our study, at the same level of VI, associations were strongest in pre-rut and fawning, emphasizing the changes in socio-spatial organisation during the mating season. Also, at the same level of VI, associations were strongest between adult females, suggesting that females are more interested in being together than are males.

Between- and within-class lagged association rate analyses indicated that classes of adult mule deer mostly disassociated, either rapidly within one day, or over longer time periods. Furthermore, it also showed that in a small proportion of associations between certain classes, the probability of re-association did not increase or decrease over time, i.e. was constant. LARs always remained above the null association rate. This suggests that if deer re-associate, they are more likely to do so with individuals of the same class as their previous associates than with individuals from the population at random [21].

There were marked sex differences in the temporal patterns of associations. Females mostly (71.1%) related to other females in long-term associations with a declining probability of re-association over time. In contrast, when females were found with males, they were mostly (72.9%) not found with males again on the next day. The long-term probability of females re-associating with other females may be explained by a greater preference for one another. The rapid splitting of female-male associations may be due to males’ brief assessments of females’ reproductive status, or to unintended encounters at specific focal points in the study area such as waterholes and grain spills. MM and YY were the only two models in which the LARs appeared cyclic (i.e. decreased and then increased). A non-exponential model, such as one with a trigonometric function of the lag (e.g. cos(t)), may work best for these two cases (pg. 206 in [21]); further testing is required.

In addition to finding that age was not a significant predictor of pairwise association strengths, we observed negligible age differences in LARs among age classes. We defined an association based on proximity obtained from GPS locations, and our study only focused on adult deer as GPS collars were too heavy to be deployed in younger (< 21 months old) individuals. If we could instead define an association based on group membership from direct observations, we might find interesting age differences in association strength and in LARs, as all age classes could be included in such analyses.

We previously reported that deer showing clinical signs of CWD were less likely to be found in groups than their healthy counterparts [25]. However, in the current study we have shown that CWD infection was not an important predictor of pairwise spatial association strengths among adult mule deer once season, year, VI, and sex of the associating pair were accounted for. As we had sufficient data on infected individuals (700/982 pairs included at least one infected deer), this finding suggests that clinical signs affect the probability of grouping, but not the proportion of time spent in association, as this was defined in our study. Fine-scale studies based on observations of direct animal to animal contacts may reveal effects of disease status on interaction rates. Interestingly though, in the LARs analysis, we found that when one or both members were CWD-negative, a proportion (~37 to 40%) of the associations had a constant probability of re-association, whereas when both members were CWD-positive, this element was not present, but rather all associations had a declining probability of re-association. The causes and consequences of these findings are unclear; however, they support that infected and non-infected deer relate to others in a different way.

In conclusion, we have increased understanding of the factors affecting the pairwise spatial association strengths of adult mule deer, and how individual characteristics such as sex, age and disease status alter the temporal stability of spatial associations among classes of individuals. This adds to both studies of behavioural ecology and of disease dynamics. Current epidemiological models of CWD are lacking empirical data on the structure of mule deer society. The association matrices generated in this study can be used in network models or individual-based spatial models that require the inclusion of more realistic (i.e. heterogeneous) data on association indices to better guide and inform disease management strategies.

## Supporting information

S1 Fig Box plots of observed association indices by season and year among adult mule deer in Saskatchewan, Canada. LG = late gestation, F = fawning, PR = pre-rut, R = rut, and EG = early gestation. Most seasons (all except PR 2010, EG 2010 and EG 2011) had a median of 0. The maximum and minimum values in every season were 1 and 0, respectively. Interquartile 3 (in dark grey) depicts values from the median to the 75th percentile. Interquartile 2 (in light grey) depicts values from the median to the 25th percentile. Mean association index is depicted with a white rhombus.
(TIF)

S2 Fig Box plots of observed association indices among pairs of adult mule deer in Saskatchewan, Canada. FF = pairs of females. FM = female-male pairs. MM = pairs of males. LG = late gestation, F = fawning, PR = pre-rut, R = rut, and EG = early gestation. Most season and sex combinations (all except FM in rut, and MM in late gestation, pre-rut and early gestation) had a median of 0. The maximum and minimum values in every season and sex combinations were 1 and 0, respectively. Interquartile 3 (in dark grey) depicts values from the median to the 75th percentile. Interquartile 2 (in light grey) depicts values from the median to the 25th percentile. Mean association index is depicted with a white rhombus.
(TIF)

S3 Fig Predicted (black) and observed (grey) pairwise association indices by sex class at increasing levels of volume of intersection (VI) among adult mule deer in Saskatchewan, Canada. FF = pairs of females, FM are female-males pairs, and MM are pairs of males. 95% confidence intervals depicted in dotted lines.
(TIF)

S4 Fig Seasonal variation of predicted (black) and observed (grey) pairwise association indices at increasing levels of volume of intersection (VI) among adult mule deer in Saskatchewan, Canada. 95% confidence intervals depicted in dotted lines.
(TIF)

S1 Appendix Datasets used to investigate factors affecting association strength (aim 1), and temporal patterns of associations (aim 2). (XLSX)

S2 Appendix Details on temporal patterns of spatial associations among mule deer in a chronic wasting disease endemic area in Saskatchewan, Canada. Tables A, B, C, D, E, F, and G; and Figures A, B, C, D, E, F, G, H, and I.
(DOCX)

S3 Appendix Analysis of variance table of top model (model 15) for strength of pairwise spatial association among adult mule deer in Saskatchewan, Canada, and variance and standard deviation of random effect (i.e. dyad). (XLSX)

S4 Appendix Model output with predicted association indices and pairwise comparisons. Tables H, I and J.
(XLSX)
