# Supplementary figures and images for: Mule deer spatial association patterns and potential implications for transmission of an epizootic disease
Source: PLoS One. 2017 Apr 7;12(4):e0175385. doi: 10.1371/journal.pone.0175385 (PMC5384682; doi:10.1371/journal.pone.0175385)

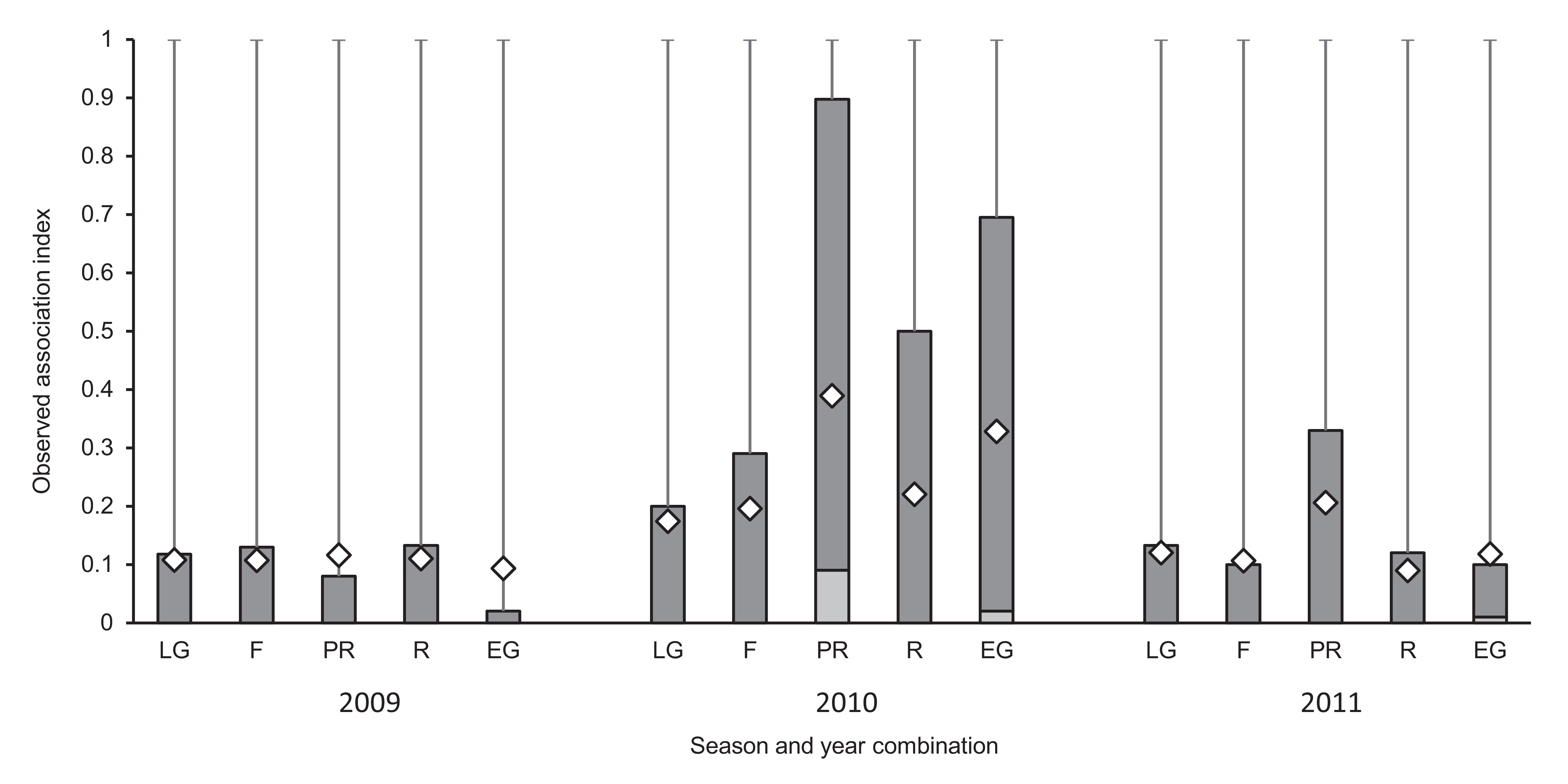

Supplement: S1 Fig — LG = late gestation, F = fawning, PR = pre-rut, R = rut, and EG = early gestation. Most seasons (all except PR 2010, EG 2010 and EG 2011) had a median of 0. The maximum and minimum values in every season were 1 and 0, respectively. Interquartile 3 (in dark grey) depicts values from the median to the 75th percentile. Interquartile 2 (in light grey) depicts values from the median to the 25th percentile. Mean association index is depicted with a white rhombus. (TIF) [file pone.0175385.s001.tif]

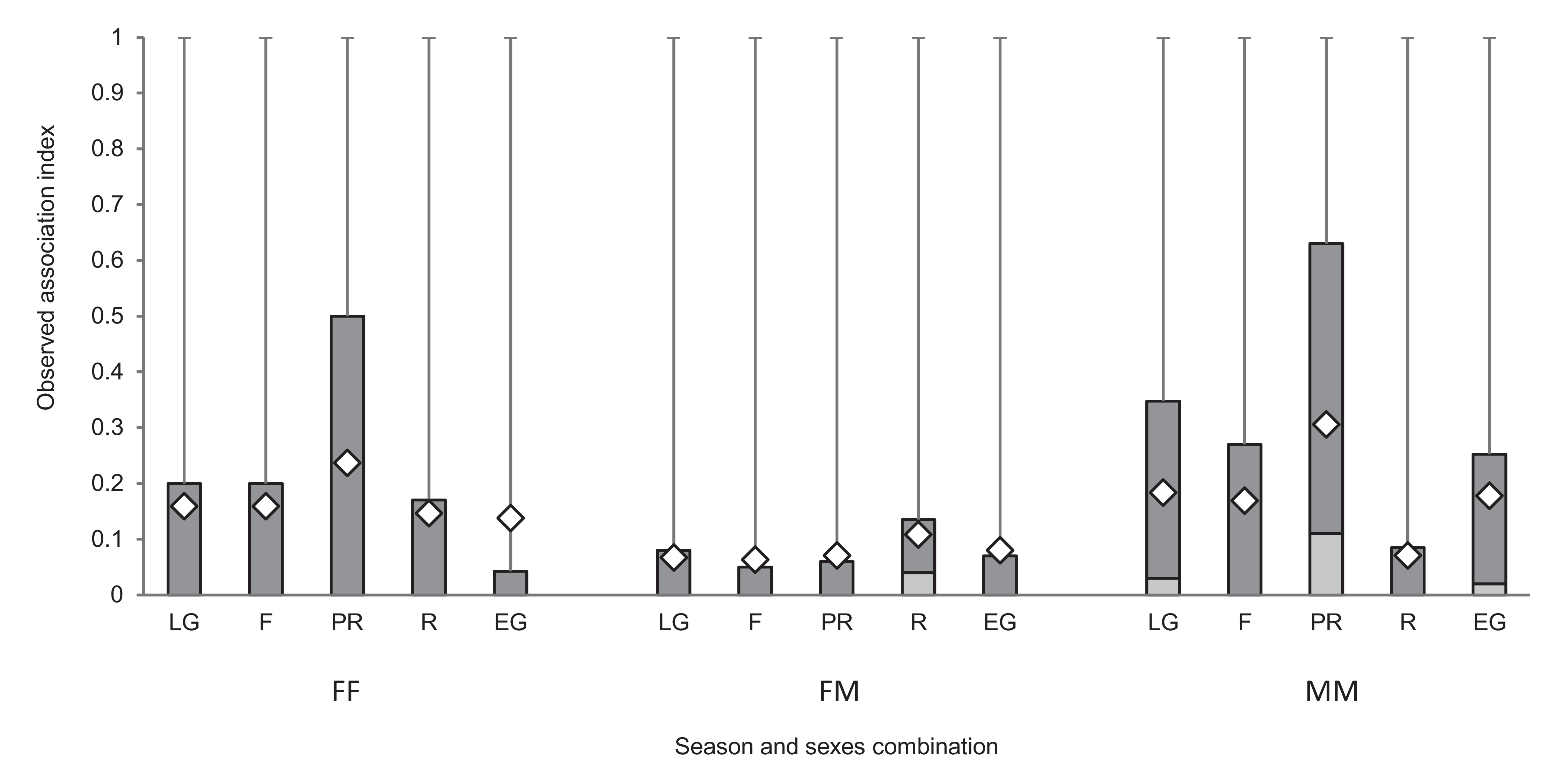

Supplement: S2 Fig — FF = pairs of females. FM = female-male pairs. MM = pairs of males. LG = late gestation, F = fawning, PR = pre-rut, R = rut, and EG = early gestation. Most season and sex combinations (all except FM in rut, and MM in late gestation, pre-rut and early gestation) had a median of 0. The maximum and minimum values in every season and sex combinations were 1 and 0, respectively. Interquartile 3 (in dark grey) depicts values from the median to the 75th percentile. Interquartile 2 (in light grey) depicts values from the median to the 25th percentile. Mean association index is depicted with a white rhombus. (TIF) [file pone.0175385.s002.tif]

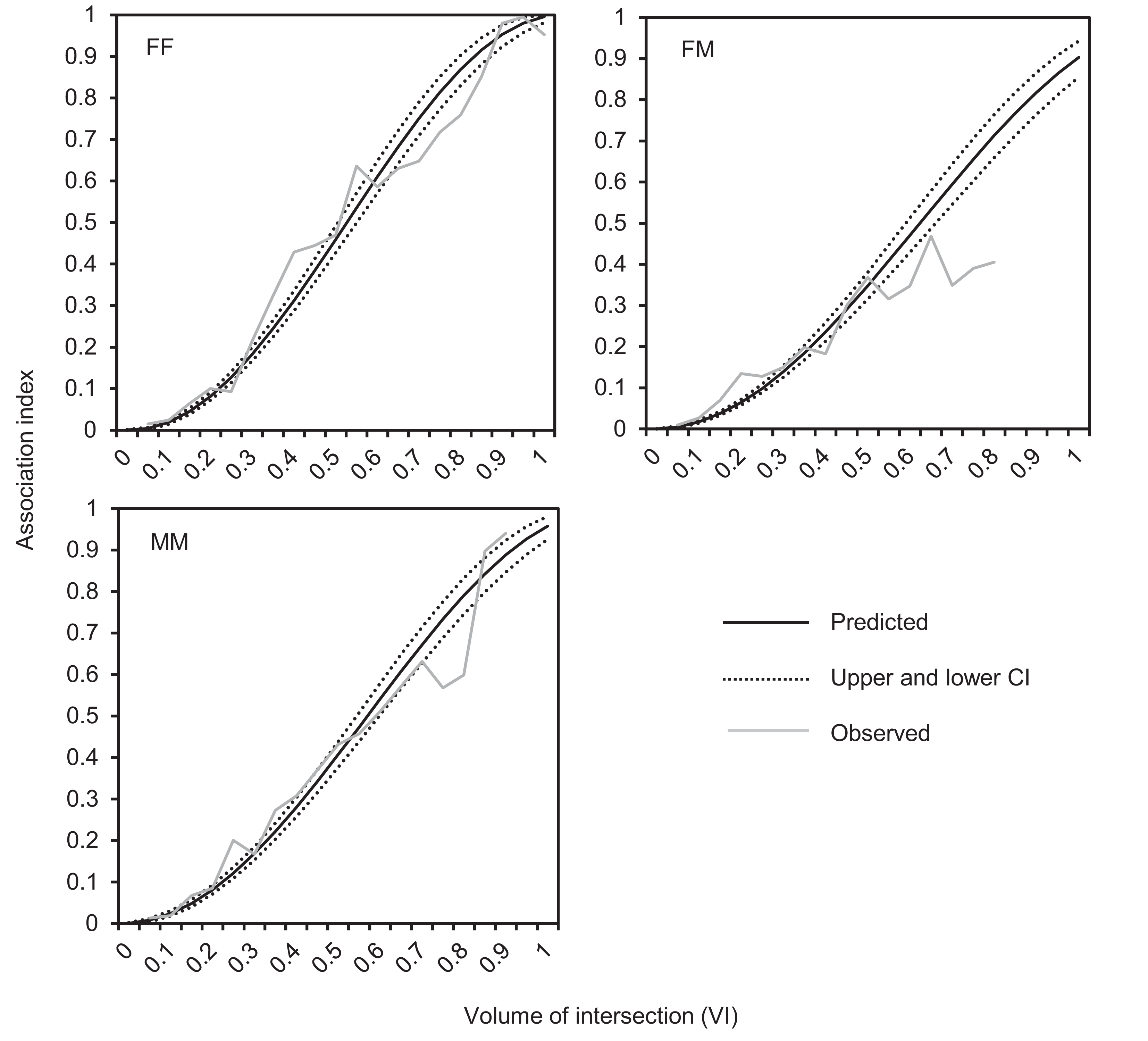

Supplement: S3 Fig — FF = pairs of females, FM are female-males pairs, and MM are pairs of males. 95% confidence intervals depicted in dotted lines. (TIF) [file pone.0175385.s003.tif]

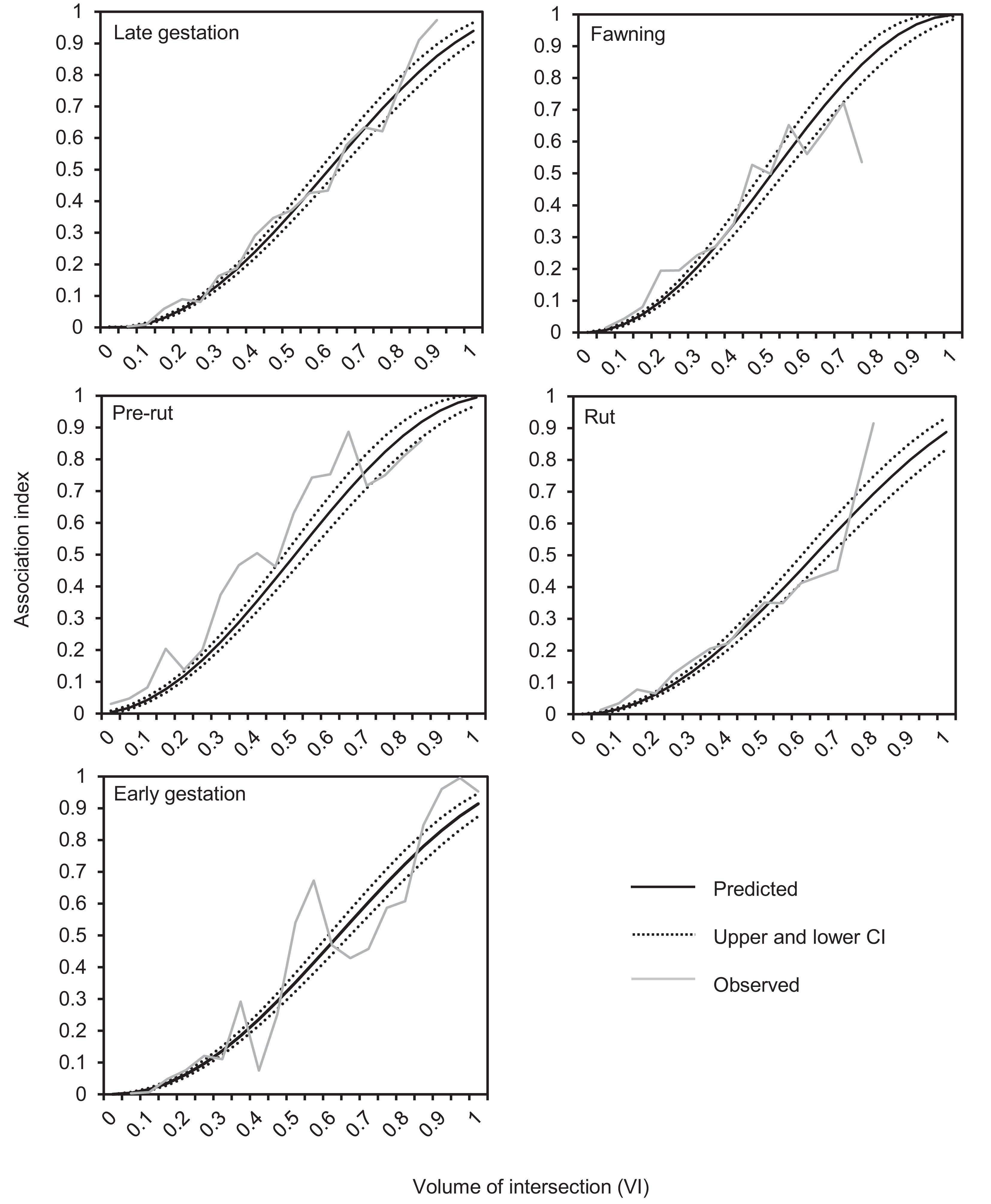

Supplement: S4 Fig — 95% confidence intervals depicted in dotted lines. (TIF) [file pone.0175385.s004.tif]
