# Supplementary material for: Mule deer spatial association patterns and potential implications for transmission of an epizootic disease
Source: PLoS One. 2017 Apr 7;12(4):e0175385. doi: 10.1371/journal.pone.0175385 (PMC5384682; doi:10.1371/journal.pone.0175385)
Supplement: S2 Appendix — Tables A, B, C, D, E, F, and G; and Figures A, B, C, D, E, F, G, H, and I. (DOCX) [file pone.0175385.s006.docx]

**S2 Appendix**

Details on temporal patterns of spatial associations among mule deer in a chronic wasting disease endemic area in Saskatchewan, Canada

To test for sex, age and chronic wasting disease (CWD) status differences in temporal stability of associations among adult Rocky Mountain mule deer (*Odocoileus hemionus hemionus*) we analysed lagged association rates (LARs) in SOCPROG 2.6 (Whitehead, 1995, 2008, 2009, 2014).

We included 44 individuals with every-2-hour GPS-telemetry data from April 1^st^ 2011 to March 31^st^ 2012 (i.e. study year 2011), and that survived the whole year.

These are the number of individuals for each sex, age and CWD status class, as well as the number of records and unique pairs in the dataset:

**Table A.** Number of mule deer individuals from different classes in the study.

| Class | Number of individuals in the study |
| --- | --- |
| Female (F) | 21 |
| Male (M) | 23 |
|  |  |
| Older (O) | 28 |
| Younger (Y) | 16 |
|  |  |
| CWD-positive (P) | 32 |
| CWD-negative (N) | 12 |

**Table B.** Number of records and unique pairs for different types of pairs in the study.

| Class | Pair class | Record count | Unique pairs count |
| --- | --- | --- | --- |
| Sex | FF | 1656 | 50 |
|  | FM | 1365 | 172 |
|  | MM | 2589 | 122 |
|  |  |  |  |
| Age | OO | 1645 | 124 |
|  | OY | 3182 | 175 |
|  | YY | 783 | 45 |
|  |  |  |  |
| CWD diagnosis | PP | 2770 | 190 |
|  | PN | 2290 | 134 |
|  | NN | 550 | 20 |
|  |  |  |  |
| Total | All | 5610 | 344 |

To calculate LARs in SOCPROG 2.6, we:

1. Set the sampling period as “date” (i.e. 1 day).
2. Defined associations as grouped in sampling period.
3. Simultaneously fitted a set of 7 mathematical models to the observed LARs. These models were of the exponential family and were composed of all, one, or any meaningful combination of three components: rapid disassociations (associations lasting 1 day at most), casual acquaintances (associations that decay over time; their average duration is approximated from the exponent of the exponential function, e.g. 1/a1, in days), and preferred/constant companionships (associations that do not decay or increase over time; their duration is interpreted within the context of the study period, in this case 1 year) (Whitehead, 1995).

**Table C.** Seven mathematical models fitted to the observed lagged association rates.

| Model ID | Model type^a^ | Quick model explanation^b^ |
| --- | --- | --- |
| 1 | a1 | Rapid dis. + pref. comps |
| 2 | exp(-a1*td) | Casual acqs |
| 3 | a2*exp(-a1*td) | Rapid dis. + casual acqs |
| A | a2+a3*exp(-a1*td) | Rapid dis. + pref. comps + casual acqs |
| 5 | a2+(1-a2)*exp(-a1*td) | Pref. comps + casual acqs |
| B | a3*exp(-a1*td)+a4*exp(-a2*td) | Rapid dis. + two levels of casual acqs |
| 7 | a3*exp(-a1*td)+(1-a3)*exp(-a2*td) | Two levels of casual acqs |
| **a** The time lag is represented by ‘td’ and the parameters of the models by ‘a1’, ‘a2’, ‘a3’ and ‘a4’. Representation of the parameters vary among models (e.g. a2 in model A is not the same as a2 in model B).  **b** Model explanations should not be taken literally without some thought as different types of social systems can produce similar patterns of lagged association rates which fit the same mathematical model (Whitehead, 2008). | | |

1. Identified, for each of the pair classes, the best fitting and most parsimonious model by the smallest quasi-Akaike information criterion (QAIC) (Whitehead, 2007).
2. Estimated error around the association rates and their durations with a jackknife procedure over 3-day periods, and in some cases (MM, YY and PP) over 30-, 45- and 10-day periods, respectively, to obtain better estimates (Whitehead, 1995).
3. Compared LARs to a null association rate (NAR), the expected LAR if animals had associated randomly, given the daily number of associations of each individual (Whitehead, 2008).

**Results**

The between-class LARs were best described by either one of two models: (A) a model containing rapid disassociations, constant companionships and casual acquaintances, or (B) a model containing rapid disassociations and two levels of casual acquaintances, one lasting longer than the other.

**Table D.** Parameters (and their standard errors) as obtained from best fitting model A.

| Class | Jackknife | a1 | a1 SE | a2 | a2 SE | a3 | a3 SE |
| --- | --- | --- | --- | --- | --- | --- | --- |
| FM | 3 | 0.057 | 0.029 | 0.130 | 0.012 | 0.141 | 0.033 |
| OO | 3 | 0.026 | 0.014 | 0.294 | 0.024 | 0.196 | 0.033 |
| OY | 3 | 0.021 | 0.011 | 0.463 | 0.020 | 0.142 | 0.025 |
| NN | 3 | 0.024 | 0.011 | 0.367 | 0.038 | 0.207 | 0.046 |
| PN | 3 | 0.031 | 0.008 | 0.399 | 0.020 | 0.187 | 0.028 |

Model A is given by the formula

a2+a3*exp(-a1*td)

where a2 is the proportion of preferred/constant companionships, a3 is the proportion of casual acquaintances, and a1 is the rate of decay of a3 (to approximate the average duration of a3, use 1/a1). The proportion of rapid disassociation is calculated by 1-a2-a3.

**Table E.** Proportions and temporal characteristics of associations as obtained from best fitting model A.

|  |  |  | Casual acquaintances | |
| --- | --- | --- | --- | --- |
| Class | % Rapid dis. | % Constant comp. (SE) | % (SE) | Duration in days (range) |
| FM | 72.9 | 13.0 (1.2) | 14.1 (3.3) | 17 (12 to 36) |
| OO | 51.1 | 29.4 (2.4) | 19.6 (3.3) | 39 (25 to 88) |
| OY | 39.5 | 46.3 (2.0) | 14.2 (2.5) | 47 (32 to 95) |
| NN | 42.6 | 36.7 (3.8) | 20.7 (4.6) | 43 (29 to 79) |
| PN | 41.4 | 39.9 (2.0) | 18.7 (2.8) | 32 (26 to 43) |

**Table F.** Parameters (and their standard errors) as obtained from best fitting model B.

| Class | Jackknife | a1 | a1 SE | a2 | a2 SE | a3 | a3 SE | a4 | a4 SE |
| --- | --- | --- | --- | --- | --- | --- | --- | --- | --- |
| FF | 100 | 0.895 | 0.532 | 0.001 | 0.000 | 0.232 | 0.141 | 0.711 | 0.052 |
| MM | 30 | 0.010 | 0.721 | -0.005 | 0.010 | 0.516 | 0.068 | 0.091 | 0.505 |
| YY | 45 | 0.027 | 0.621 | -0.002 | 0.004 | 0.254 | 0.224 | 0.243 | 0.182 |
| PP | 10 | 0.538 | 0.397 | 0.001 | 0.000 | 0.293 | 0.169 | 0.499 | 0.046 |

Model B is given by the formula

a3*exp(-a1*td)+a4*exp(-a2*td)

where a3 is the proportion of casual acquaintances of shorter duration, a4 is the proportion of casual acquaintances of longer duration, a1 is the rate of decay of a3 (to approximate the average duration of a3, use 1/a1), and a2 is the rate of decay of a4 (to approximate the average duration of a4, use 1/a2). The proportion of rapid disassociation is calculated by 1-a3-a4.

**Table G.** Proportions and temporal characteristics of associations as obtained from best fitting model B.

|  |  | Shorter casual acquaintances | |  | Longer casual  acquaintances | |
| --- | --- | --- | --- | --- | --- | --- |
| Class | % Rapid dis. | % (SE) | Duration in days (range) |  | % (SE) | Duration in days (range) |
| FF | 5.7 | 23.2 (14.1) | 1 (1 to 3) |  | 71.1 (5.2) | 980 (662 to 1889) |
| MM | 39.3 | 51.6 (6.8) | 96 (1 to 1) |  | 9.1 (50.5) | 182 (67 to 248) |
| YY | 50.3 | 25.4 (22.4) | 37 (2 to 2) |  | 24.3 (18.2) | 514 (173 to 530) |
| PP | 20.8 | 29.3 (16.9) | 2 (1 to 7) |  | 49.9 (4.6) | 1062 (721 to 2121) |

**Figures A to E. Lagged association rate (in blue), null association rate (in red) and the best fit model (in green), for different sex, age and CWD classes of adult mule deer (*Odocoileus hemionus hemionus*) monitored from 1 April 2011 to 31 March 2012, in Antelope Creek, Saskatchewan, Canada.** The best fit model included rapid disassociations, constant companions and casual acquaintances. Bars are jackknifed standard errors.

| Figure A. Female-male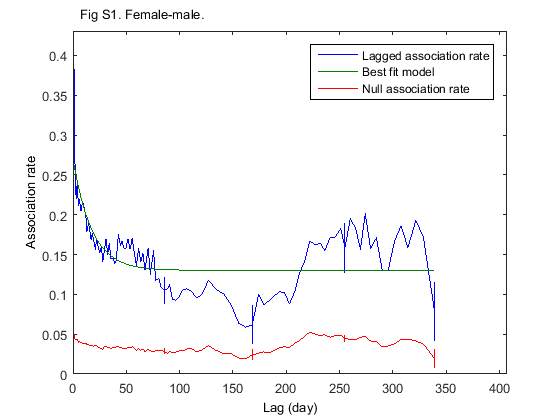 |
| --- |
| Figure B. Old-old.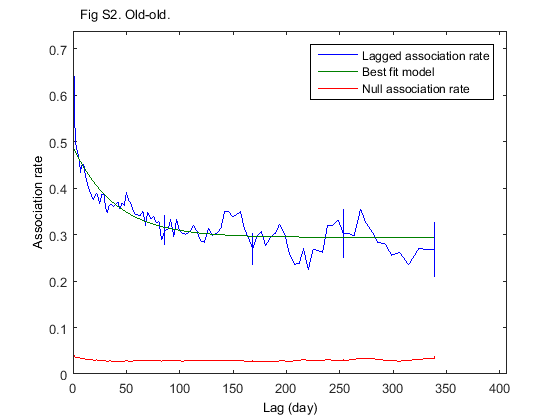 |
| Figure C. Old-young.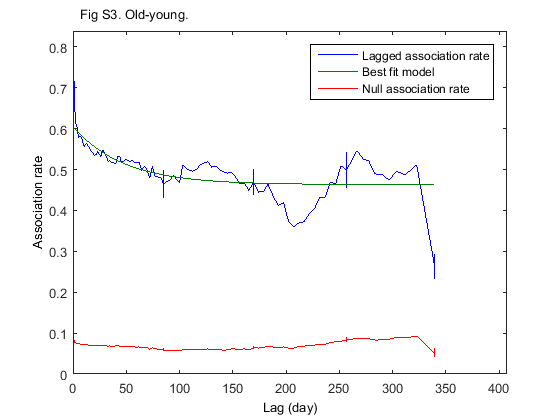 |
| Figure D. Negative-negative.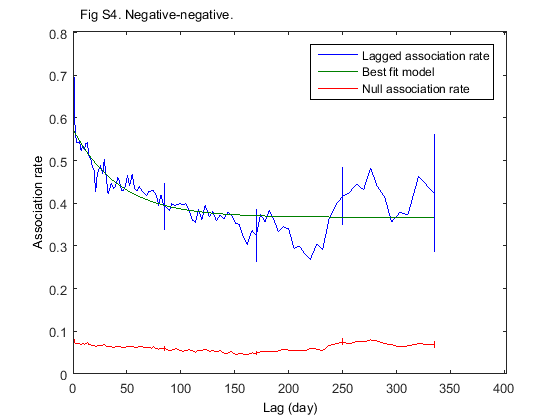 |
| Figure E. Positive-negative. 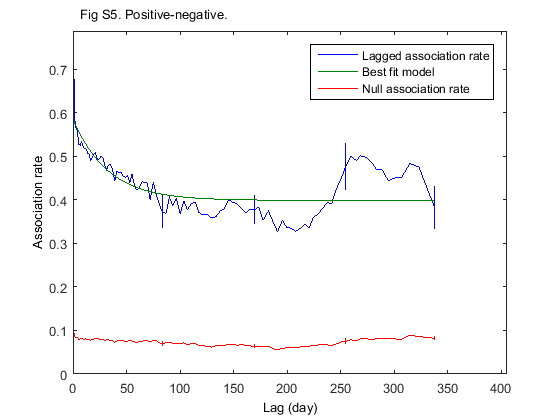 |

**Figures F to I. Lagged association rate (in blue), null association rate (in red) and the best fit model (in green), for different sex, age and CWD classes of adult mule deer (*Odocoileus hemionus hemionus*) monitored from 1 April 2011 to 31 March 2012, in Antelope Creek, Saskatchewan, Canada.** The best fit model included rapid disassociations, and two levels of casual acquaintances. Bars are jackknifed standard errors.

| Figure F. Female-female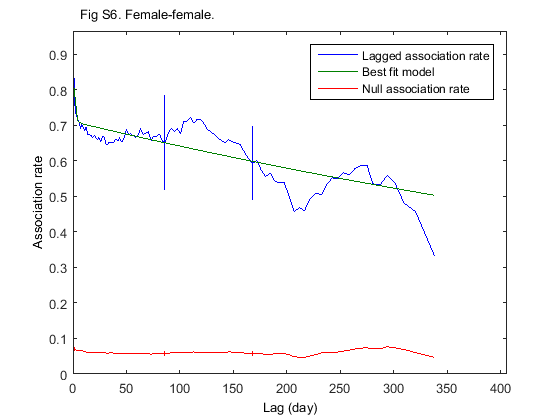 |
| --- |
| Figure G. Male-male.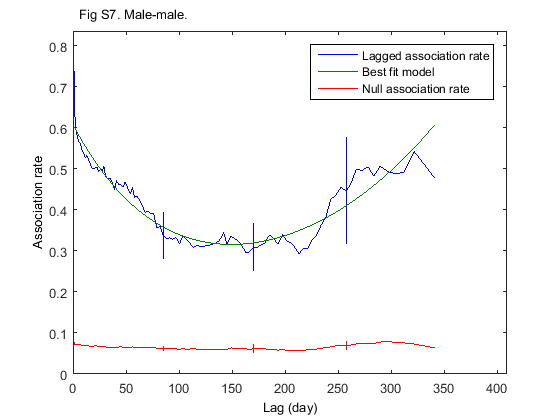 |
| Figure H. Young-young.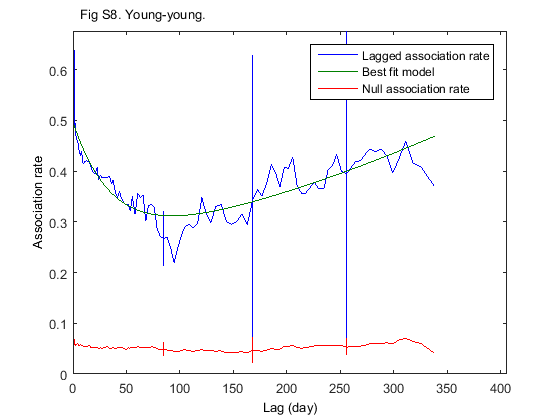 |
| Figure I. Positive-positive. 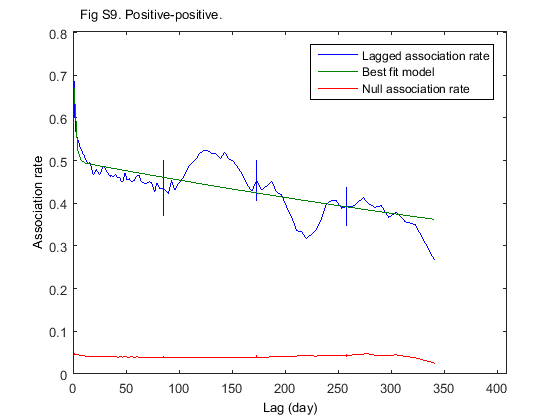 |

Whitehead, H. (1995). Investigating structure and temporal scale in social organizations using identified individuals. *Behavioral Ecology, 6*(2), 199-208. doi: 10.1093/beheco/6.2.199

Whitehead, H. (2007). Selection of models of lagged identification rates and lagged association rates using AIC and QAIC. *Communications in Statistics - Simulation and Computation, 36*(6), 1233-1246. doi: 10.1080/03610910701569531

Whitehead, H. (2008). *Analyzing animal societies. Quantitative methods for vertebrate social analysis*. USA: The University of Chicago Press.

Whitehead, H. (2009). SOCPROG programs: analyzing animal social structures. *Ecology and Sociobiology, 63*, 765-778.

Whitehead, H. (2014, April 2014). *SOCPROG programs: analyzing animal social structures - SOCPROG2.5 (release 2014a)*. Halifax, Nova Scotia, Canada.
